# Catatonic Schizophrenia Associated With Cerebrospinal GAD65 Autoantibodies: Case Report and Literature Review

**DOI:** 10.3389/fimmu.2022.829058

**Published:** 2022-02-09

**Authors:** Niels Hansen, Claudia Bartels, Bianca Teegen, Jens Wiltfang, Berend Malchow

**Affiliations:** ^1^Department of Psychiatry and Psychotherapy, Medical University Center Göttingen, Göttingen, Germany; ^2^Euroimmun Reference Laboratory, Luebeck, Germany; ^3^German Center for Neurodegenerative Diseases (DZNE), Göttingen, Germany; ^4^Neurosciences and Signaling Group, Institute of Biomedicine (iBiMED), Department of Medical Sciences, University of Aveiro, Aveiro, Portugal

**Keywords:** catatonia, GAD65 autoantibodies, schizophrenia, autoimmunity, psychiatry

## Abstract

**Background:**

GAD65 autoimmunity is reported to be associated with schizophrenia and bipolar disorder. However, there has been no evidence that glutamic acid decarboxylase 65 (GAD65) autoantibodies in cerebrospinal fluid (CSF) are associated with akinetic catatonia in schizophrenia patients.

**Methods:**

We report the case of a 28-year-old man who underwent diagnostics including brain MRI, neuropsychological testing, and electroencephalography (EEG) as well as a tumor search *via* CT of the abdomen and thorax, as well as colonoscopy and gastroscopy. For clinical characterization, his patient files were retrospectively examined.

**Results:**

Our patient presented catatonia that responded somewhat to benzodiazepines in combination with previously taken antipsychotics such as risperidone for prediagnosed paranoid schizophrenia. Diagnostics revealed GAD65 autoantibodies in his serum and CSF. MRI revealed no brain lesion, and the tumor search had no malignancy. We diagnosed catatonic schizophrenia. Furthermore, as he had not fully recovered, he was given immunotherapy entailing two cycles of intravenous immunoglobulins. Subsequent neuropsychological testing due to subjective cognitive complaints after immunotherapy revealed no objective cognitive deficits.

**Conclusions:**

We present the novel finding of an association between GAD65 autoantibodies in the serum and CSF with catatonia in a patient suffering from prediagnosed chronic schizophrenia. Due to the presence of CSF GAD65 antibodies and the catatonia factor in prediagnosed schizophrenia, we suspect that his catatonia has an autoimmune origin. Immunotherapy stabilized the catatonia that had initially responded to lorazepam treatment. Further research should be done to characterize patients’ responses to immunotherapy and standard treatment in a large cohort of patients with GAD65 antibody-associated catatonia and schizophrenia.

## Introduction

According to the latest *Diagnostic and Statistical Manual of Mental Disorders*, fifth edition (DSM V), catatonia is considered an independent disease entity (1, 2). It is a clinical psychomotor syndrome manifesting in either an excited (hyperkinetic) or retarded (akinetic) form. The latter is characterized by immobility, mutism, and rigidity, often coinciding with clinical features like abnormal posturing, echolalia or echopraxia, and waxy flexibility, whereas the hyperkinetic form reveals psychomotor agitation (1). Autoimmune catatonia is a catatonia subtype, recently characterized by Rogers et al. (3). Specific cell-membrane surface autoantibodies have been linked to catatonia, such as those against *N*-methyl-d-aspartate (NMDA) (4–14), gamma-aminobutyric acid A (GABAA) receptor (15–17) (**Table 1**), and voltage-gated potassium channels (8) or myelin (20) (for an overview, see **Table 1**).

**Table 1:** Overview of the diversity of neural autoantibody-associated catatonia.

Number of patients	Psychiatric disease	Auto-antibodies	References
189/347	Depression,	NMDAR	(5)
	psychosis		(6)
	Anxiety		(7)
	Cognitive dysfunction		(8)
	Delirium		(9)
	Behavioral		(10)
	abnormalities		(11)
	Mania		(12)
			(4)
			(18)
			(13)
			(14)
3/41	Behavioral	GABAAR	(15)
	changes		(16)
			(17)
1/12	Depression	VGKC	(8)
	Anxiety		
	Suicidality		
1/1	Psychosis	Unknown	(19)
		epitope,	
		somatodendritic	
		staining	
10/20	Schizophrenia	Myelin	(20)

GABAAR, gamma-aminobutyric acid receptor A; NMDAR, N-methyl-d-aspartate receptor; VGKC, voltage-gated potassium channel.

Catatonia has not yet been described to be associated with glutamic acid decarboxylase 65 (GAD65) autoantibodies, but GAD65 antibodies are known to occur in schizophrenia. An analysis of pooled data from 9 different cohort studies revealed that GAD65 antibodies are detected in 4.6% of patients with schizophrenia compared with 2.7% in healthy controls with no relevant differences (21). However, our analysis also revealed that psychotic patients carry twice the risk of developing GAD65 autoantibodies than controls (21). GAD65 is a catalyzing enzyme in GABA synthesis. Its blockage by GAD65 antibodies led to motor dysfunction in an animal model, caused by impaired GABAergic transmission (22), and to motor dysfunction in stiff-person syndrome (23).

GAD65 antibodies are thus likely to cause motor dysfunction manifesting as catatonia, although there is no evidence to date that GAD65 autoantibodies play a causal role. Here, we present the first case of GAD65 antibodies in the serum and cerebrospinal fluid (CSF) of a patient with schizophrenia who suffered catatonia responding to benzodiazepine and later immunotherapeutic treatment with intravenous immunoglobulins (IVIGs).

## Case Report

**Figure 1:**
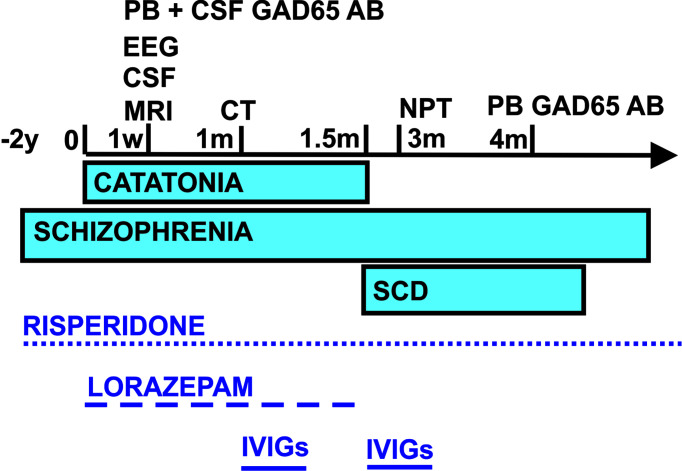
Schematic overview of the time course regarding symptom presentation, diagnostics, and treatment. CSF, cerebrospinal fluid; EEG, electroencephalography; GAD65 AB, glutamic acid decarboxylase autoantibodies; IVIGs, intravenous immunoglobulins; m, month; NPT, neuropsychological testing; PB, peripheral blood; SCD, subjective cognitive decline; w, week; y, years.

We present the case of a 28-year-old male who presented for the first time in our Department of Psychiatry and Psychotherapy, University Medical Center Göttingen, with akinetic catatonia initially presenting as a stupor and immobility (**Figure 1**). The patient has a master’s in bioengineering after studying for 5 years in India. He is a native Hindi and English speaker and migrated to Germany in 2020 to start a PhD in neuroscience. He attended school for 12 years in India. His roommate found him immobile on the floor, failing to respond to any speech. The roommate called the emergency medical services to have him transported to our department. After being given midazolam, he was able to start speaking. Midazolam has been given in addition to the antipsychotic medications he was already taking [risperidone (4 mg/day)]. He was later treated with 5.5 mg/day of lorazepam, and his stupor resolved somewhat within hours after taking lorazepam. He reported that he had paranoia (being watched) before the catatonia started, indicative of psychotic symptoms of his pre-diagnosed paranoid schizophrenia 2 years ago in India (treated with risperidone) (**Figure 1**). Apart from his risperidone therapy, he had undergone no medical interventions in the past. Furthermore, his psychosocial history revealed no abnormalities. No somatic comorbidities were reported.

His psychopathology revealed a growing sensation of being watched by other people (mild delusions of reference). We were unable to explore any delusions or hallucinations or ego disturbances. The sensation of being observed by other people became weaker within 2 weeks. His neurological examination was unremarkable, and no paresis was apparent. However, he did describe a feeling of stiffness that was less pronounced at night and not present in the neurological examination. His patient history revealed a prior episode of catatonia in 2014 while traveling by train. At that time, he was also hungry and stressed out.

Differential diagnosis (**Figure 1**) *via* brain MRI revealed no pathological brain lesions. Electroencephalography (EEG) early in his time course exhibited no epileptic potentials or focal or multifocal slowing. No continuous EEG monitoring was done. CSF analysis *via* lumbar puncture showed no pleocytosis or elevated protein levels. Furthermore, we detected no intrathecal IgG synthesis. CSF analysis also yielded anti-GAD65 autoantibodies in immunoblots (1:100), and his blood analysis revealed GAD65 autoantibodies in immunoblots (1:3,200) and rodent immunohistochemistry (**Table 2** and **Figure 1**). We searched for anti-Zic4, anti-TR, anti-SOX1, anti-Ri, anti-Yo, anti-Hu, anti-amphiphysin, and anti-CV2/CRMP5 antibodies *via* immunoblots. Cell-based assays involving indirect immunohistochemistry were done to search for anti-NMDAR, anti-Leucin Rich Glioma Inactivated Protein 1 (LGI1), anti-amino-3-hydroxy-5-methyl-4-isoxazolepropionic acid receptor 1/2 (AMPAR1/2), anti-gamma-aminobutyric acid receptor 1/2 (GABABR1/2), anti-dipeptidyl-peptidase-like 6 protein (DPPX), and anti-contactin associated protein 2 (CASPR2) antibodies.

**Table 2:** Laboratory and clinical parameters.

Laboratory parameters	First presentation	Follow-up
**Antibodies**		
GAD65 serum	1:3,200	1:3200
GAD65 CSF	1:100	–
**Cells CSF**		
Cells/µl (<5 µg/L)	0	–
Lymphocytes %	80	–
Monocytes %	14	–
Lactat mmol/L	1.4	–
**Proteins CSF**		
Total protein mg/L	319	–
Albumin mg/L	216	–
IgG mg/L	29.4	–
IgA mg/L	2.2	–
IgM mg/L	0.17	–
QAlb %	5.3	–
QIgG %	3	–
QIgA %	1.6	–
QIgM %	0.3	–
**CSF blood–brain barrier disturbance**	No	–
**CSF intrathecal IgG synthesis**	No	–
**Neuropsychological and clinical parameters**		
MMSE (screening) orientation	10/10	–
CDT (screening)	01	–
TMT Part A (processing speed)	50–50	–
WAIS-IV Coding (processing speed)	25	–
TAP Alertness (phasic alertness) Index	58	–
TAP Divided Attention (divided attention), Omissions	24	–
TAP Go/NoGo (selective attention), errors	58	–
TMT Part B (flexibility)	40–50	–
RWT semantic fluency—alternating (flexibility)	29	–
RWT letter fluency—alternating (flexibility)	52	–
RCFT Copy (visual functions)	≥16	–
WAIS-IV Block Design (visuoconstruction)	63	–
WAIS-IV Digit Span forward (memory span)	91	–
WAIS-IV Digit Span backward (working memory)	50	–
RAVLT Sum trials 1–5 (verbal learning)	50	–
RAVLT trial 7 (verbal delayed recall)	70–85	–
RAVLT trials 5–7 (verbal long-term memory/retention)	65–85	–
WMS-IV Visual Reproduction I (visual immediate recall)	16	–
WMS-IV Visual Reproduction II (visual delayed recall)	35	–

For neuropsychological parameters starting from TMTA to WMS-IV, percentage ranges are indicated.

Alb, albumin; CERAD, Consortium to Establish a Registry for Alzheimer’s Disease; CDT, Clock Drawing Test; IgA, immunoglobulin A; IgG, immunoglobulin G; IgM, immunoglobulin M; LTM, long-term memory; M, memory; MMSE, Mini Mental Status Examination; P-Tau 181, phosphorylated tau protein 181; Q, quotient; RCFT, Rey Complex Figure Test; RWT, Regensburg Word Fluency Test; TAP, test of attentional performance; TMT, Trail Making Test; WAIS-IV, Wechsler Adult Intelligence Scale, fourth edition; WMS-IV, Wechsler Memory Scale, fourth edition.

Three weeks after his initial presentation, he was sent to the Neurology Department, University Medical Center Göttingen, and given IVIGs (160 g) because of incompletely resolved symptoms and remaining stiffness, psychotic symptoms, and spatial disorientation. During the 3 weeks in the Department of Psychiatry and Psychotherapy (with his first stay in a protected and later non-protected setting), lorazepam was slowly reduced and finally stopped after IVIG application because his symptoms were alleviated on a psychiatric ward after his stay in the Department of Neurology, University Medical Center Göttingen (**Figure 1**). IVIGs were chosen for this patient as his initial treatment regimen because corticosteroids would have had the potential to exacerbate psychotic symptoms or induce depressive symptoms. CT of the thorax and abdomen performed during his stay in the Department of Neurology, University Medical Center Göttingen, revealed no malignancy, nor did the colonoscopy and gastroscopy (**Figure 1**). His gastroscopy showed a reflux esophagitis grade A (Los Angeles classification). One and a half months later, he received IVIGs again to stabilize his current status, as his symptoms had not disappeared entirely (**Figure 1**). Immediately after his catatonic episode terminated, he reported persistent cognitive decline at regularly scheduled outpatient visits, namely, difficulty finding words and memory deficits, especially recalling conversations. He also complained of losing things and felt like he was losing control of his own actions. We therefore had him undergo extensive neuropsychological testing 3 months after his initial presentation (done in English) (**Figure 1**). In all cognitive tests, including screening tests and tests of attention, executive, visuoconstructive/visual, and memory functions, his performance was normal (**Table 2**). Only the immediate recall of visual stimuli was weaker than could be expected from his premorbid level but was still within the normal range (low average). Therefore, we classified his cognitive complaints as subjective cognitive decline. Additional autoantibody testing 4 months later revealed again anti-GAD65 autoantibodies in serum (1:3,200) (**Figure 1**). Our patient is still taking risperidone regularly at a dosage of 4 mg/day. Stupor together with rigidity and immobility that responds to benzodiazepine is diagnosed as akinetic catatonia most likely caused by an underlying chronic paranoid schizophrenia; his illness is thus referred to as catatonic schizophrenia associated with serum and CSF GAD65 autoantibodies. We assumed that his catatonia had an immune origin, as we had detected CSF GAD65 autoantibodies in a psychotic patient in conjunction with a hypokinetic movement disorder symptom like catatonia, which was in line with autoimmune catatonia or autoimmune psychosis with catatonia according to recent guidelines from Pollak et al. (24) and Hansen et al. (25). As another supporting criterion for a catatonia’s autoimmune origin, we detected responsivity to immunotherapy. We had originally suspected stiff-person syndrome as the differential diagnosis, but he failed to meet the criteria for that diagnosis, such as a hyperlordosis or pronounced stiffness. Due to his normal EEG, a persistent non-convulsive status is unlikely, although we cannot rule it out entirely as he did not undergo continuous EEG monitoring. Moreover, we observed no indications of temporal lobe seizures or other seizure types like musicogenic epilepsy, which is reportedly associated with GAD65 autoantibodies (26–28). His EEG revealed no epileptic potentials, making seizures of a temporal origin or otherwise unlikely, although they cannot be fully excluded. As no bitemporal signal abnormalities were found in MRI, his EEG was unremarkable, no temporal lobe epilepsy was diagnosed, and a definitive GAD65-positive limbic encephalitis is highly unlikely.

## Discussion

To our knowledge, it is a novel finding that we detected CSF GAD65 antibodies in association with catatonia, in particular, catatonic schizophrenia. GAD65 antibodies have been reported by many investigators in conjunction with schizophrenia (21), at a higher frequency than in control subjects. GABAergic signaling might be implicated in schizophrenia, as studies have shown weaker GAD67 (29) and GAD65 expression (30) in the brain tissue of schizophrenic patients. Another hint originates from evidence showing that a GABA-related protein like GAD65 is known to be downregulated in the MK-801 schizophrenia-like model in mice, with consequences for GABA metabolism and homeostasis (31). It is thus likely that GAD65 antibodies that impair GABAergic neuronal signaling might affect the disease activity in patients with schizophrenia. Furthermore, catatonia might originate from heightened neuronal activity in premotor regions, as Walther et al. reported (32). They showed that it is unclear whether such increased activity in premotor regions is attributable to locally heightened neural activity *via* cortico-cortical inhibition or inhibitory transmission in cortical-basal ganglia circuits. GAD65 antibodies might conceivably disturb GABAergic signaling in both neuronal loops, resulting in catatonia. A recent study (33) revealed that catatonia correlated with increased functional connectivity in cerebellum-dependent networks and decreased oscillations in a low-frequency band in basal ganglia networks. Interestingly, the same study demonstrated that motor functions (evident in motor scores correlating strongly with functional network activity in cortico-striatal connections) add support to the second hypothesis of Walther et al. (32) with the most prominent anomalies in neural activity occurring in cortico-basal ganglia loops. The planning and generation of movements are triggered by a complex interplay between excitatory (mostly glutamatergic) and inhibitory GABAergic signaling in motor regions. It is therefore not surprising that autoimmune catatonia might be associated with neural autoantibodies against the two most involved receptors, such as excitatory glutamatergic NMDAR on the one hand (4–14) and inhibitory GABAAR on the other (15–17) (**Table 1**). Immunotherapy *via* corticosteroids, immunoglobulins, and rituximab alleviated catatonia in these patients (4, 15). In GABAAR-positive catatonia, immunotherapy proved effective in treating catatonia, unlike lorazepam and antipsychotics (15). In our case, his catatonia had already responded to benzodiazepine application, which was supplemented by adding IVIGs, underlying the possibility of different response patterns depending on the type of associated antibody. GAD65 antibodies target an intracellular epitope, making it less probable that these autoantibodies mediate the catatonia, as a T cell-driven pathomechanism is often detected in GAD65 autoimmunity (i.e., limbic encephalitis) (34). In contrast, GABAR antibodies are more probably involved in the pathogenesis of catatonia, as they are antibodies against extracellular targets. In addition to GABAAR and NMDAR antibodies, novel neural autoantibodies (against an unknown epitope in granule cells in cerebellar and hippocampal interneurons) were identified in a patient suffering from psychosis and severe chronic catatonia (19). That patient responded to immunotherapy, but not to standard treatment with antipsychotics and benzodiazepines, supporting the relevance of taking CSF and blood samples to look for both known and novel autoantibodies in order to treat these patients adequately. The strength of our case is that we have expanded the phenotypic spectrum of GAD65 autoimmunity. Limitations concern the evidence level of a case report and the absence of additional CSF parameters underlying an immunogenic origin of catatonia in our patients such as a pleocytosis of intrathecal IgG synthesis. Furthermore, long-term EEG monitoring would have facilitated an epilepsy-related GAD65 autoimmunity phenomenon if present. As GAD65 antibodies are not themselves pathogenic, a more straightforward approach would have helped us detect *via* cell flow cytometry immune cell subsets such as T cells in GAD65 autoimmunity or other inflammation parameters like neopterin. We believe that our findings deserve attention, as they provide evidence toward a novel path for investigating psychotic disorders *via* biomarkers like GAD65 autoantibodies, known to be the second most frequently detected serum autoantibody, i.e., in a large cohort of 22,472 suspected paraneoplastic and autoimmune encephalitis patients (35).

We report the case of a schizophrenic patient suffering from akinetic catatonia associated with CSF GAD65 antibodies responsive to immunotherapy. Our case reveals the possibility that GAD65 antibodies are involved in catatonia’s pathogenesis and highlights the urgency of seeking antibodies in these patients, keeping in mind its rarity, as reported in a recent study by Hoffmann et al. (36), and especially atypical GAD-related syndromes (37) as our patient’s. A positive response to standard treatment and immunotherapy has the potential to enable a good long-term outcome and maintain the alleviation of symptoms.

## Data Availability Statement

The raw data supporting the conclusions of this article will be made available by the corresponding author.

## Ethics Statement

Ethical review and approval were not required for the study on human participants in accordance with the local legislation and institutional requirements. The patient provided their written informed consent for publication.

## Author Contributions

NH wrote the manuscript. CB, JW, and BM have revised the manuscript for important intellectual content. All authors listed have made a substantial, direct, and intellectual contribution to the work and approved it for publication.

## Funding

Funding was received from the Open Access fund of the University of Göttingen.

## Conflict of Interest

BT was employed by Euroimmun Reference Laboratory.

The remaining authors declare that the research was conducted in the absence of any commercial or financial relationships that could be construed as a potential conflict of interest.

## Publisher’s Note

All claims expressed in this article are solely those of the authors and do not necessarily represent those of their affiliated organizations, or those of the publisher, the editors and the reviewers. Any product that may be evaluated in this article, or claim that may be made by its manufacturer, is not guaranteed or endorsed by the publisher.

## References

[1] Rasmussen AS Mazurek MF Rosebush PI. Catatonia: Our Current Understanding of Its Diagnosis, Treatment and Pathophysiology. World J Psychiatry (2016) 6:391–8. doi: 10.5498/wjp.v6.i4.391 PMC5183991 28078203

[2] Gazdag G Takács R Ungvari GS. Catatonia as a Putative Nosological Entity: A Historical Sketch. World J Psychiatry (2017) 7:177–83. doi: 10.5498/wjp.v7.i3.177 PMC5632602 29043155

[3] Rogers JP Pollak TA Blackman G David AS. Catatonia and the Immune System: A Review. Lancet Psychiatry (2019) 6:620–30. doi: 10.1016/S2215-0366(19)30190-7 PMC7185541 31196793

[4] Warren N Swayne A Siskind D O’Gorman C Prain K Gillis D. Serum and CSF Anti-NMDAR Antibody Testing in Psychiatry Affiliations. J Neuropsychiatry Clin Neurosci (2020) 32:154–60. doi: 10.1176/appi.neuropsych.19030079 31530118

[5] Dalmau J Gleichman AJ Hughes EG Rossi JE Peng X Lai M. Anti-NMDA-Receptor Encephalitis: Case Series and Analysis of the Effects of Antibodies. Lancet Neurol (2008) 7:1091–8. doi: 10.1016/S1474-4422(08)70224-2 PMC2607118 18851928

[6] Tsutsui K Kanbayashi T Tanaka K Boku S Ito W Tokunaga J. Anti-NMDA-Receptor Antibody Detected in Encephalitis, Schizophrenia, and Narcolepsy With Psychotic Features. BMC Psychiatry (2012) 12:37. doi: 10.1186/1471-244X-12-37 22569157 PMC3436772

[7] Desena A Graves D Warnack W Greenberg BM. Herpes Simplex Encephalitis as a Potential Cause of Anti-N-Methyl-D-Aspartate Receptor Antibody Encephalitis: Report of 2 Cases. JAMA Neurol (2014) 71:344–6. doi: 10.1001/jamaneurol.2013.4580 24473671

[8] Kruse JL Lapid MI Lennon VA Klein CJ O’ Toole O Pittock SJ. Psychiatric Autoimmunity: N-Methyl-D-Aspartate Receptor IgG and Beyond. Psychosomatics (2015) 56:227–41. doi: 10.1016/j.psym.2015.01.003 25975857

[9] Duan BC Weng WC Lin KL Wong LC Li ST Hsu MH. Variations of Movement Disorders in Anti-N-Methyl-D-Aspartate Receptor Encephalitis: A Nationwide Study in Taiwan. Med (Baltimore) (2016) 95:e4365. doi: 10.1097/MD.0000000000004365 PMC5402545 27631202

[10] Granata T Matricardi S Ragona F Freri E Zibordi F Andreetta F. Pediatric NMDAR Encephalitis: A Single Center Observation Study With a Closer Look at Movement Disorders. Eur J Paediatr Neurol (2018) 22:301–7. doi: 10.1016/j.ejpn.2018.01.012 29396169

[11] Herken J Prüss H. Red Flags: Clinical Signs for Identifying Autoimmune Encephalitis in Psychiatric Patients. Front Psychiatry (2017) 8:. doi: 10.3389/fpsyt.2017.00025 PMC5311041 28261116

[12] Restrepo-Martínez M Chacón-González J Bayliss L Ramírez-Bermúdez J Fricchione GL Espinola-Nadurille M. Delirious Mania as a Neuropsychiatric Presentation in Patients With Anti-N-Methyl-D-Aspartate Receptor Encephalitis. Psychosomatics (2020) 61:64–9. doi: 10.1016/j.psym.2019.03.002 31000140

[13] Ryan SA Costello DJ Cassidy EM Brown G Harrington HJ Markx S. Anti-NMDA Receptor Encephalitis: A Cause of Acute Psychosis and Catatonia. J Psychiatr Pract (2013) 19:157–61. doi: 10.1097/01.pra.0000428562.86705.cd 23507817

[14] Barry H Hardiman O Healy DG Keogan M Moroney J Molnar JP. Anti-NMDA Receptor Encephalitis: An Important Differential Diagnosis in Psychosis. Br J Psychiatry (2011) 199:508–9. doi: 10.1192/bjp.bp.111.092197 21984802

[15] Samra K Rogers J Mahdi-Rogers M Stanton B. Catatonia With GABA(A) Receptor Antibodies. Pract Neurol (2020) 20:139–43. doi: 10.1136/practneurol-2019-002388 31771952

[16] Pettingill P Kramer HB Coebergh JA Pettingill R Maxwell S Nibber A. Antibodies to GABAA Receptor α1 and γ2 Subunits: Clinical and Serologic Characterization. Neurology (2015) 84:1233–41. doi: 10.1212/WNL.0000000000001326 PMC4366091 25636713

[17] Nikolaus M Knierim E Meisel C Kreye J Prüss H Schnabel D. Severe GABAA Receptor Encephalitis Without Seizures: A Paediatric Case Successfully Treated With Early Immunomodulation. Eur J Paediatr Neurol (2018) 22:558–62. doi: 10.1016/j.ejpn.2018.01.002 29396172

[18] Restrepo-Martinez M Ramirez-Bermudez J Bayliss L Espinola-Nadurille M. Characterisation and Outcome of Neuropsychiatric Symptoms in Patients With Anti-NMDAR Encephalitis. Acta Neuropsychiatr (2020) 32:92–8. doi: 10.1017/neu.2019.46 31753060

[19] Endres D Rauer S Pschibul A Süß P Venhoff N Runge K. Novel Antineuronal Autoantibodies With Somatodendritic Staining Pattern in a Patient With Autoimmune Psychosis. Front Psychiatry (2020) 11:627:627. doi: 10.3389/fpsyt.2020.00627 32848899 PMC7424063

[20] Rimón R Ahokas A Ruutiainen J Halonen P. Myelin Basic Protein Antibodies in Catatonic Schizophrenia. J Clin Psych (1986) 47:26–8. doi: 10.1111/pcn.12543 2416737

[21] Grain R Lally J Stubbs B Malik S LeMince A Nicholson TR. Autoantibodies Against Voltage-Gated Potassium Channel and Glutamic Acid Decarboxylase in Psychosis: A Systematic Review, Meta-Analysis, and Case Series. Psychiatry Clin Neurosci (2017) 71:678–89. doi: 10.1111/pcn.12543 28573688

[22] Hansen N Grünewald B Weishaupt A Colaço M:N Toyka KV Sommer C. Human Stiff Person Syndrome IgG-Containing High-Titer Anti-GAD65 Autoantibodies Induce Motor Dysfunction in Rats. Experimental Neurology (2013) 239:202–9. doi: 10.1016/j.expneurol.2012.10.013 23099416

[23] Bernardo F Rebordão L André Rêgo A Sara Machado S João Passos J Costa C. Stiff Person Spectrum Disorders: An Illustrative Case Series of Their Phenotypic and Antibody Diversity. J Neuroimmunol (2020) 341:577192. doi: 10.1016/j.jneuroim.2020.577192 32087460

[24] Hansen N Lipp M Vogelgsang J Vukovich R Zindler T Luedecke D. Autoantibody-Associated Psychiatric Symptoms and Syndromes in Adults: A Narrative Review and Proposed Diagnostic Approach. Brain Behav Immun Health (2020) 9:100154. doi: 10.1016/j.bbih.2020.100154 34589896 PMC8474611

[25] Pollak TA Lennox BR Müller S Benros ME Prüss H Tebartz van Elst L. Autoimmune Psychosis: An International Consensus on an Approach to the Diagnosis and Management of Psychosis of Suspected Autoimmune Origin. Lancet Psychiatry (2020) 7:93–108. doi: 10.1016/S2215-0366(19)30290-1 31669058

[26] Smith KM Zalewski NL Budhram A Britton JW So E Cascino GD. Musicogenic Epilepsy: Expanding the Spectrum of Glutamic Acid Decarboxylase 65 Neurological Autoimmunity. Epilepsia (2021) 62:e76–81. doi: 10.1111/epi.16888 33764529

[27] Hansen N Widman G Witt JA Wagner J Becker AJ Elger CE. Seizure Control and Cognitive Improvement *via* Immunotherapy in Late Onset Epilepsy Patients With Paraneoplastic Versus GAD65 Autoantibody-Associated Limbic Encephalitis. Epilepsy Behav (2016) 65:18–24. doi: 10.1016/j.yebeh.2016.10.016.E 27855355

[28] Hansen N Ernst L Rüber T Widman G Becker AJ Elger CE. Pre- and Long-Term Postoperative Courses of Hippocampus-Associated Memory Impairment in Epilepsy Patients With Antibody-Associated Limbic Encephalitis and Selective Amygdalohippocampectomy. Epilepsy Behav (2018) 79:93–9. doi: 10.1016/j.yebeh.2017.10.033 29253681

[29] Sershen H Guidotti A Auta J Drnevich J Grayson DR Veldic M. Gene Expression Of Methylation Cycle And Related Genes In Lymphocytes And Brain Of Patients With Schizophrenia And Non-Psychotic Controls. Biomark Neuropsychiatry (2021) 5:. doi: 10.1016/j.bionps.2021.100038 PMC8341034 34368786

[30] Purves-Tyson TD Brown AM Weissleder C Rothmond DA Weickert CS. Reductions in Midbrain GABAergic and Dopamine Neuron Markers Are Linked in Schizophrenia. Mol Brain (2021) 14:96. doi: 10.1186/s13041-021-00805-7 34174930 PMC8235806

[31] Wang X Hu Y Liu W Ma Y Chen X Xue T. Molecular Basis of GABA Hypofunction in Adolescent Schizophrenia-Like Animals. Neural Plast (2021) 2021:9983438. doi: 10.1155/2021/9983438 33936193 PMC8062182

[32] Walther S Stegmayer K Wilson JE Heckers S. Structure and Neural Mechanisms of Catatonia. Lancet Psychiatry (2019) 6:610–9. doi: 10.1016/S2215-0366(18)30474-7 PMC6790975 31196794

[33] Sambataro F Hirjak D Fritze S Kubera KM Northoff G Calhoun VD. Intrinsic Neural Network Dynamics in Catatonia. Hum Brain Mapp (2021) 42:6087–98. doi: 10.1002/hbm.25671 PMC8596986 34585808

[34] Langenbruch L Bleß L Schulte-Mecklenbeck A Sundermann B Brix T Elger CE. Blood and Cerebrospinal Fluid Immune Cell Profiles in Patients With Temporal Lobe Epilepsy of Different Etiologies. Epilepsia (2020) 61:e153–8. doi: 10.1111/epi.16688 32893887

[35] Kunchok A McKeon A Zekeridou A Flanagan EP Dubey D Lennon VA. Autoimmune/Paraneoplastic Encephalitis Antibody Biomarkers: Frequency, Age, and Sex Associations. Mayo Clin Proc (2021) 23:S0025–6196(21)00651-0. doi: 10.1016/j.mayocp.2021.07.023 34955239

[36] Hoffmann C Zong S Mané-Damas M Stevens J Malyavantham K Küçükali Cİ. The Search for an Autoimmune Origin of Psychotic Disorders: Prevalence of Autoantibodies Against Hippocampus Antigens, Glutamic Acid Decarboxylase and Nuclear Antigens. Schizophr Res (2021) 228:462–71. doi: 10.1016/j.schres.2020.12.038 33581586

[37] Lacruz Ballester L Fernandez-Fournier M Puertas Muñoz I Rodriguez Fraga O Fernandez-Escandon CL Rodriquez de Rivera Garrido FJ. Serum Glutamate Decarboxylase Antibodies and Neurological Disorders: When to Suspect Their Association? Neurol Sci (2020) 43:633–41. doi: 10.1007/s10072-021-05281-4 33914193

